# Patient-Reported Symptoms after Midfacial Trauma

**DOI:** 10.1055/s-0041-1742174

**Published:** 2022-01-17

**Authors:** Nina Pauli, Martina Grinups, Lena Folkestad, Gunnhildur Gudnadottir

**Affiliations:** 1 Department of Otorhinolaryngology, Institute of Clinical Sciences, Sahlgrenska Academy at the University of Gothenburg, Sahlgrenska University Hospital, Gothenburg, Sweden

**Keywords:** midfacial fracture, facial trauma, patient-reported outcome

## Abstract

**Background** The aim of this study was to assess patient-reported symptoms and health-related quality of life, 12 to 24 months after injury in patients with midfacial fractures.

**Methods** Patients diagnosed with midfacial fractures were assessed regarding symptoms related to the fracture as well as assessment of the patients overall health-related quality of life using the Gothenburg Trismus Questionnaire (GTQ), the Folkestad facial trauma questionnaire, and EuroQol five-dimensional (EQ-5D). Questionnaires were distributed to the study patients 12 to 24 months after the trauma. Medical records were retrospectively surveyed for age, gender, trauma etiology, date of injury, fracture classification, treatment regimen, and time of surgery.

**Results** Sixty-seven percent of the study group reports sensibility disturbance in the face 12 to 24 months after trauma and 52% reported cosmetic consequences related to the trauma. Numbness in the face was the symptom reported to be most disturbing for the patients. Few of the patients reported severe jaw-related problems, problems with muscular tension, or eating limitation according to the validated questionnaire GTQ.

**Conclusion** Sensibility disturbance remains a significant and common symptom 12 to 24 months after midfacial trauma. There is a need for a validated patient-reported outcome instrument for facial trauma that covers multiple aspects of facial trauma such as vision disturbance and diplopia, jaw-related problems, and facial pain as well as sensibility disturbance and cosmetic consequences.

Maxillofacial trauma can lead to both acute and more long-term symptoms, such as vision disturbance and enophthalmos, jaw-related problems, malocclusion, and sensibility disturbance. In addition, maxillofacial injuries can be associated with cosmetically disturbing facial deformities and scars and can have a negative psychological impact. 1 2 3 4

For many decades, research within the field of maxillofacial trauma has focused mostly on surgical outcome measured objectively or assessed by a doctor/surgeon. However, over the last years there is an increasing amount of studies taking the patients' experience into account using patient-reported outcome (PRO) instruments to evaluate symptoms and health-related quality of life (HRQL) after facial trauma. 5 6 7 8

In several fields of medical research the introduction of PRO instruments has revealed differences in the patients' and the doctors' point of view regarding symptom burden and treatment outcomes. 9 As a case in point, the prevalence of sensory disturbance after maxillofacial trauma has differed substantially (7–64%) depending on research methodology and methods of assessment. 10 11 Thus, illustrating the need for PRO in clinical trials and also in clinical practice to enable direct input from the patients in a systematic manner.

To our experience there are no existing validated PRO instrument addressing and covering the different symptoms after facial trauma. In this study, we have introduced the validated Gothenburg Trismus Questionnaire (GTQ) earlier used in head and neck cancer (HNC) and in addition an instrument earlier used in patients with facial trauma and orbital floor fractures, as well as the HRQL instrument EuroQol five-dimensional (Eq. 5D).

## Aim

The aim of this study was to assess patient-reported symptoms and HRQL, 12 to 24 months after injury in patients with midfacial fractures.

## Material and Methods

### Study Protocol and Data Collection

Patients diagnosed with midfacial fractures at the ear, nose, and throat department at a tertiary referral center in Sweden during 2014 were identified using the International Statistical Classification of Diseases and Related Health Problems 10th Revision (ICD-10) diagnose coding system, from the outpatient registry. 12

### Inclusion and Exclusion Criteria

Inclusion criteria were: age > 18 years, midfacial fractures according to ICD-10 (including the following; S02.0 Fracture of vault of skull, S02.3 Fracture of orbital floor, S02.4 Fracture of malar and maxillary bones, S02.7 Multiple fractures involving skull and facial bones, S02.8 Fractures of other skull and facial bones and, S02.9 Fracture of skull and facial bones, part unspecified). Exclusion criteria were: isolated skull base fractures, nasal bone fractures, and fractures of the mandible.

Medical records were retrospectively surveyed for age, gender, trauma etiology, date of injury, fracture classification, treatment regimen, and time of surgery. Surgical approach as well as surgical method and any orbital floor implant and/or osteosynthesis material used was noted in the study protocol.

### Fracture Classification

Fractures were classified based on the computed tomography scans as follows:

I. Isolated zygomatic arch fracture
  (a) Not displaced (< 3 mm)
  (b) Displaced (> 3 mm)
II. Orbital floor fracture
III. Medial orbit fracture
IV. Le Fort fracture
V. Zygomaticomaxillary fractures
  (a) Not displaced (< 3 mm)
  (b) Displaced (> 3 mm)
  (c) Multifragment

### Patient-Reported Outcome

Questionnaires regarding symptoms related to the midfacial fracture as well as assessment of the patients' overall HRQL were distributed to the study patients 12 to 24 months after the trauma. All nonresponders were reminded once.

### Folkestad Facial Trauma Questionnaire

This is a facial trauma-specific questionnaire including items assessing vision disturbance, cosmetic consequence from injury, sensibility disturbance, and jaw-related problems after facial trauma. The questionnaire has earlier been used and described in studies on orbital floor fractures. 13 14

### Gothenburg Trismus Questionnaire

The GTQ is a validated, symptom-specific questionnaire focusing on trismus, facial pain, jaw-related problems, and muscular tension in HNC. The GTQ consists of 21 items and 3 main domains (jaw-related problems, eating limitations, and muscular tension). GTQ score ranges from 0 to 100, where 100 indicates the maximal amount of symptoms, and 0 indicate no symptoms. The GTQ has been previously used for patients with HNC and patients with temporomandibular disorder (TMD). 15

### EQ-5D

The EQ-5D is a well-known and widely used questionnaire assessing HRQL. EQ-5D evaluates five dimensions of health and an overall rating of the patient's experience of health. 16 17

## Ethics

The study was approved by the Regional Ethical Review Board at Gothenburg University and performed in accordance with the Declaration of Helsinki. All study subjects gave their informed consent to participate.

## Statistical Methods

Descriptive data are presented with mean and standard deviation when applicable. For comparison between study group and lost-to follow-up group, the Fisher's exact test (lowest one-sided *p* -value multiplied by 2) was used for dichotomous variables. The Mantel–Haenszel chi-square exact test was used for ordered categorical variables and chi-square exact test was used for nonordered categorical variables. The Mann–Whitney *U* test was used for continuous variables.

## Results

### Study Group and Lost to Follow-Up

**Fig. 1:**
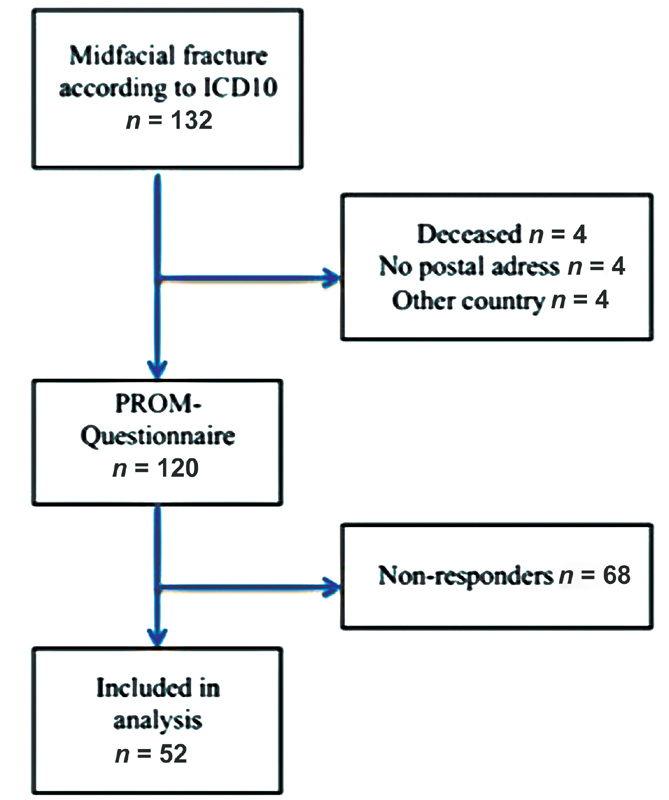
Study flowchart.

A total of 132 patients were identified with midfacial fracture according to the ICD-10 during the study year. Out of these, postal address was possible to identify in 120 patients to whom questionnaires was sent out to. Fifty-two patients responded and were available for further analysis ( Fig. 1 ).

Analysis of the lost to follow-up group revealed a significantly lower mean age among the nonresponders ( *p* -value 0.038), furthermore, patients experiencing interpersonal violence were more likely to be nonresponders ( *p* -value 0.044). We found no other significant differences between the groups.

### Patient and Fracture Characteristics

A majority of the study patients were men, 62%. Zygomaticomaxillary complex fractures were the most common type of midfacial fracture in our material. Sixty-two percent of the fractures were displaced (> 3 mm) or multifragment. Fall accidents were the dominating cause of accident followed by sports accident and bicycle accident ( Table 1 ).

**Table 1:** Patient characteristics, fracture classification, and cause of injury

	Study patients *n* = 52
Gender	*n* (%)
Female	20 (39)
Male	32 (61)
Age	
Mean, y (min-max)	47 (18–91)
Type of fracture	*n* (%)
Zygomaticomaxillary	32 (62)
Orbital floor	9 (17)
Le Fort	5 (10)
Isolated zygomatic arch	3 (6)
Medial orbit	2 (4)
Other	1 (2)
Severity of fracture	
Not displaced	20 (38)
Displaced	15 (29)
Multifragment	17 (33)
Cause of injury	
Fall accident	21 (40)
Sport accident	12 (23)
Bicycle accident	8 (15)
Interpersonal violence	7 (14)
Hit by accident	2 (4)
Other/unknown	1 (2)
Motor vehicle accident	1 (2)

Regarding surgical treatment subciliary incision was the most common approach for fracture treatment. Gillies incision as well as frontozygomatic suture incision was also commonly used. A majority (86%) of the patients who needed surgical reposition of fracture were treated within 14 days from the trauma. Osteosynthesis material was used in the majority of the surgically treated cases ( Table 2 ).

**Table 2:** Fracture treatment information: surgical procedure, approach, and surgical material

Time of surgery	*n* (%)
No surgery	24 (46)
Surgery within 7 d	11 (21)
Surgery 8–14 d	13 (25)
Surgery > 15 d	4 (8)
Open/closed reduction	*n* = 28
Closed reduction	5 (18)
Open reduction	23 (82)
Surgical approach a	*n* = 28
Subciliary incision	17 (61)
Transconjunctival incision	2 (7)
Frontozygomatic suture incision	7 (25)
Intraoral incision	5 (18)
Through existing wound	2 (7)
Gillies incision	8 (29)
Bicoronal flap	1 (4)
Material used in surgery	
Titanium plates and screws for osteofixation	16 (57)
Porous polyethylene orbital floor implants b	11 (39)

a Multiple approaches can be used depending on the type of fracture.

b Medpor *n* = 7, Synpor with titanium mesh *n* = 3.

### Patient-Reported Outcome

**Fig. 2:**
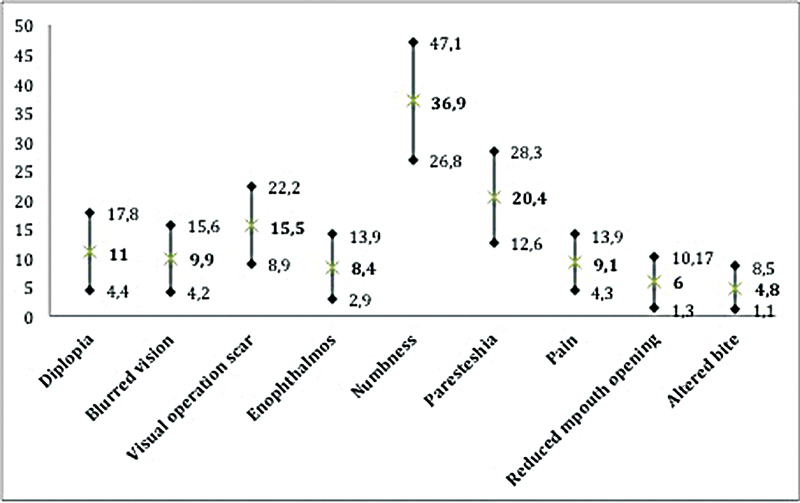
Impact of patient-reported symptoms after midfacial trauma. How often symptoms are disturbing, Visual Analogue Scale (VAS) score in mm (0–100) where 0 means never disturbing and 100 means always disturbing. Mean and 95% confidence interval (CI).

More than two-thirds, 67%, of the study group reports sensibility disturbance in the face 12 to 24 months after trauma and more than half of the investigated patients, 52%, reported cosmetic consequences related to the trauma. Among the cosmetic aspects after facial trauma a visual facial scar was the most disturbing complaint. Furthermore, sensibility disturbances in the teeth were reported by 40% of the study patients ( Table 3 ). Numbness in the face was the symptom reported to be most disturbing for the patients ( Fig. 2 ).

**Table 3:** Patient-reported symptoms, prevalence of symptoms, *n* (%)

	Total, *n* = 52 (%)	Surgery, *n* = 28 (%)	No surgery *n* = 24 (%)
Vision disturbance	14 (27)	9 (32)	5 (21)
Cosmetic consequence	27 (52)	17 (61)	10 (42)
Sensibility disturbance	35 (67)	21 (75)	14 (58)
Mouth opening/bite affected	12 (23)	8 (29)	4 (17)
Abnormal sensibility of teeth	21 (40)	14 (50)	7 (29)

Rather few of the patients reported severe jaw-related problems, problems with muscular tension, or eating limitation according to the validated questionnaire GTQ. For reference, mean score for patients with TMD, patients with radiation-induced trismus, and healthy controls are presented in Table 4 . Separate analysis with regards to jaw-related problems and trismus was performed excluding orbital floor fractures, since patients with orbital floor fractures are not expected to develop trismus. The analyses revealed very small differences and are therefore reported in text only.

**Table 4:** GTQ mean score and standard deviation for patients with midfacial fractures

GTQ Mean (SD)	Study patients *n* = 51	HNC *n* = 78	TMD *n* = 51	Controls a , *n* = 129
Jaw-related problems	9,2 (17.0)	43.5	73.2	5.1
Eating limitation	9.4 (17.6)	45.0	52.2	1.3
Muscular tension	16.2 (19.7)	20.4	54.0	13.0

Abbreviations: GTQ, Gothenburg Trismus Questionnaire; HNC, head and neck cancer; SD, standard deviation; TMD, temporomandibular disorder.

Note: Reference values for other study populations from Johnson et al. Development and validation of the Gothenburg Trismus Questionnaire (GTQ), 9 used with permission. Domains and single items range from 0 to 100 where 100 means maximal amount of symptoms and 0 is equal to no symptoms.

a Healthy controls.

For assessment of HRQL according to EQ-5D, 52% of the study group reported problem with pain or discomfort and 29% reported problem with anxiety or depression ( Table 5 ).

**Table 5:** EQ-5D-3L health-related quality of life for patients with midfacial fractures

	No problems, *n* (%)	Problems, *n* (%)	Population norm data a Problems %
Mobility	48 (92)	3 (6)	8.6
Self-care	49 (94)	2 (4)	1.5
Activity	42 (80)	9 (17)	7.9
Pain/discomfort	24 (46)	27 (52)	40.8
Anxiety/depression	36 (69)	15 (29)	26.0
	Mean (SD), median		
Overall health a	77.4 (23.2), 88		83.3

Abbreviations: EQ-5D, EuroQol five-dimensional; SD, standard deviation; VAS, Visual Analogue Scale.

a Population norm data for Sweden from the EuroQoL group. 16

b VAS score 0–100, 100 = best imaginable health, 0 = worst imaginable health.

## Discussion

We found that long-term sequelae after facial trauma is common and that sensibility disturbance was rated as the most disturbing symptom for the patients in this study on patient-reported symptoms 1 to 2 years after midfacial trauma.

The most common cause of trauma was fall accident in this study. Causes of injury vary a lot between different countries and the impact of late sequelae on HRQL can vary depending on the cause of injury. Earlier studies have shown that violence as the cause of injury is a risk factor for a prolonged period of convalescence and a later return to work after trauma compared with other causes of injury, as well as an increased risk of depressive symptoms. 18 19

In this study, patients who were lost to follow-up were younger and to a larger extent exposed to interpersonal violence or assault compared with the study patients. This suggests that the result of the present study might be an underestimation of the actual symptom burden.

A large European research project on zygomatic fractures and mandibular fractures revealed that assault was the most common cause of injury and that predominately men are affected by maxillofacial trauma. 20 21

Patient who underwent surgery reported more problems with persistent symptoms after 1 to 2 years compared with patients with fractures that were treated conservatively in this study. This is in consistency with other studies. The reason for this is multifactorial and can partially be explained by the fact that most of the surgically treated patient suffered from more complex and dislocated fractures than those treated conservatively. 22

### Patient-Reported Outcome

We found that sensibility disturbance is a common long-term sequel for facial trauma patients, in this study reported by more than two-thirds of the patients. Sensibility disturbance was graded as the most disturbing symptom 1 to 2 years after trauma.

Many studies have undertaken objective assessments for sensibility disturbance but the correlation with patient-reported symptoms are poor and risks to underestimate the problem. 23 For example, Souyris et al investigated 1,394 cases of midfacial fractures and found the prevalence of sensibility disturbance to be 7.2% when assessed by the surgeon, 11 whereas Sakavicius et al found a prevalence of 64.4% when using both objective testing and patient-reported assessment. 10 Folkestad and Granstrom compared the doctor's assessment and patient's experience of symptoms after facial trauma (using separate protocols for doctor and patient, at five occasions during 1 year) and showed that there is a discrepancy between the patients' and the doctors' experience above all when it comes to sensibility disturbance. 14

All in all, the present study supports that the use of PRO is important in evaluation of sensibility disturbance and that sensibility disturbance is of great significance for the patients even though sometimes overlooked by clinicians and regarded as a mild symptom.

The second most common long-term sequel in this study was cosmetic impact from visual facial scar. More than half of the patients in the study confirmed symptoms with cosmetic impact. A study by Tebble et al showed that even smaller facial laceration can have a long-term impact on the patient and that the impact is related to both type of trauma and the patient's level of emotional distress. Tebble et al showed that injuries caused by assault was associated with more problems with cosmetic consequences and psychological distress. 24 Another study by Rahtz et al on appearance concern and trauma suggested in the same way that the cosmetic impact of trauma is correlated to the patient's general level of psychological distress and not specifically related to the location of the trauma. 25

We found no differences in anxiety and depression compared with the norm population data according to the HRQL instrument EQ-5D as one could have expected given the facial trauma. Again, patients exposed to interpersonal violence were underrepresented in our material and might have revealed another picture.

In this study, the prevalence of trismus and jaw-related problems was low. When comparing the results from the study patients with HNC patients and TMD patients, patients with midfacial fractures scored very low and more in line with a healthy population. Very few studies have focused on trismus and jaw-related problems after midfacial trauma. Chang et al studied patients with zygomaticomaxillary complex fractures with and without involvement of the temporomandibular joint (TMJ) and found that trismus is very common preoperatively both when the joint is involved and not, but a majority of the patients improves after surgery. 26 Folkestad and Granstrom found a significant difference between the doctor's assessment and patient's experience of restricted mouth opening both preoperatively and at 1 month after surgery. There was no TMJ involvement in any of these cases. 14

Facial trauma is described as one of the etiological factors to the development of TMD but it seems that that the risk of more longstanding jaw-related problems is evident primarily when the mandible joint or TMJ is directly involved in the fracture. 27 28

Regarding the PRO instruments introduced in this study we conclude that the GTQ, which is a symptom-specific and validated instrument, could not as a whole detect the patients' main problems especially with sensibility disturbances. The Folkestad facial trauma questionnaire that has earlier been used in studies on orbital floor fracture is relevant but needs validation and psychometric testing in a larger patient cohort.

### Study Limitations

A limitation of this study is the cross-sectional design where baseline data and symptoms pre- and postoperatively cannot be assessed. The dropout rate was high in terms of nonresponders to the questionnaires used in the study.

## Conclusion

Sensibility disturbance remains a significant and common symptom 12 to 24 months after midfacial trauma. The use of PRO instrument is warranted but also challenging in this group of patients. There is a need for a validated PRO instrument for facial trauma that covers multiple aspects of facial trauma such as vision disturbance and diplopia, jaw-related problems, and facial pain as well as sensibility disturbance and cosmetic consequences.

## References

[1] Back C PN McLean N R Anderson P J David D J The conservative management of facial fractures: indications and outcomes J Plast Reconstr Aesthet Surg 2007 60 02 146 151 17223512 10.1016/j.bjps.2006.01.032

[2] Lozada K Kadakia S Abraham M T Ducic Y Complications of midface fractures Facial Plast Surg 2017 33 06 557 561 29195235 10.1055/s-0037-1607447

[3] Ukpong D I Ugboko V I Ndukwe K C Gbolahan O O Health-related quality of life in Nigerian patients with facial trauma and controls: a preliminary survey Br J Oral Maxillofac Surg 2008 46 04 297 300 18336970 10.1016/j.bjoms.2007.09.013

[4] Prashanth N T Raghuveer H P Kumar D Shobha E S Rangan V Rao T S Anxiety and depression in facial injuries: a comparative study J Int Oral Health 2015 7 09 94 100 PMC4589729 26435626

[5] Kaukola L Snäll J Roine R Sintonen H Thorén H Health-related quality of life of patients with zygomatic fracture Med Oral Patol Oral Cir Bucal 2017 22 05 e636 e642 28809377 10.4317/medoral.21914 PMC5694188

[6] Sharma G Kaur A Quality of life after orbito-facial trauma Orbit 2017 36 06 407 410 28812415 10.1080/01676830.2017.1337204

[7] Conforte J J Alves C P Sánchez M P Ponzoni D Impact of trauma and surgical treatment on the quality of life of patients with facial fractures Int J Oral Maxillofac Surg 2016 45 05 575 581 26723500 10.1016/j.ijom.2015.11.022

[8] Ologunde R McLeod N MH Use of patient-reported outcome measures in oral and maxillofacial trauma surgery: a review Br J Oral Maxillofac Surg 2018 56 05 371 379 29650475 10.1016/j.bjoms.2018.03.010

[9] Fayers P M Quality of Life the Assessment, Analysis and Interpretation of Patient-Reported Outcomes 2nd ed. Chichester John Wiley & Sons, Ltd. 2007

[10] Sakavicius D Juodzbalys G Kubilius R Sabalys G P Investigation of infraorbital nerve injury following zygomaticomaxillary complex fractures J Oral Rehabil 2008 35 12 903 916 19090908 10.1111/j.1365-2842.2008.01888.x

[11] Souyris F Klersy F Jammet P Payrot C Malar bone fractures and their sequelae. A statistical study of 1.393 cases covering a period of 20 years J Craniomaxillofac Surg 1989 17 02 64 68 2921331 10.1016/s1010-5182(89)80047-2

[12] World Health Organization ICD-10: International Statistical Classification of Diseases and Related Health Problems: Tenth Revision 2nd ed. Geneva World Health Organization 2004

[13] Folkestad L Westin T Long-term sequelae after surgery for orbital floor fractures Otolaryngol Head Neck Surg 1999 120 06 914 921 10352449 10.1016/S0194-5998(99)70336-0

[14] Folkestad L K Granstrom G Patients' experiences and doctors' opinions on recovery after surgery for orbital fractures Otolaryngol Head Neck Surg 2004 131 02 254 255

[15] Johnson J Carlsson S Johansson M Development and validation of the Gothenburg Trismus Questionnaire (GTQ) Oral Oncol 2012 48 08 730 736 22418239 10.1016/j.oraloncology.2012.02.013 PMC5479349

[16] EuroQol Group EuroQol–a new facility for the measurement of health-related quality of life Health Policy 1990 16 03 199 208 10109801 10.1016/0168-8510(90)90421-9

[17] Brooks R EuroQol: the current state of play Health Policy 1996 37 01 53 72 10158943 10.1016/0168-8510(96)00822-6

[18] Borgna S C Klein K Harvey L E Batstone M D Factors affecting return to work following facial trauma Plast Reconstr Surg 2013 132 06 1525 1530 24005371 10.1097/PRS.0b013e3182a8069d

[19] Rahtz E Bhui K Smuk M Hutchison I Korszun A Violent injury predicts poor psychological outcomes after traumatic injury in a hard-to-reach population: an observational cohort study BMJ Open 2017 7 05 e014712 10.1136/bmjopen-2016-014712 PMC5777458 28559457

[20] Brucoli M Boffano P Broccardo E The “European zygomatic fracture” research project: the epidemiological results from a multicenter European collaboration J Craniomaxillofac Surg 2019 47 04 616 621 30765246 10.1016/j.jcms.2019.01.026

[21] Brucoli M Boffano P Pezzana A The “European Mandibular Angle” research project: the analysis of complications after unilateral angle fractures Oral Surg Oral Med Oral Pathol Oral Radiol 2019 128 01 14 17 30981529 10.1016/j.oooo.2019.02.027

[22] Kloss F R Stigler R G Brandstätter A Complications related to midfacial fractures: operative versus non-surgical treatment Int J Oral Maxillofac Surg 2011 40 01 33 37 20870393 10.1016/j.ijom.2010.08.006

[23] Neovius E Fransson M Persson C Clarliden S Farnebo F Lundgren T K Long-term sensory disturbances after orbitozygomatic fractures J Plast Reconstr Aesthet Surg 2017 70 01 120 126 27769603 10.1016/j.bjps.2016.09.007

[24] Tebble N J Thomas D W Price P Anxiety and self-consciousness in patients with minor facial lacerations J Adv Nurs 2004 47 04 417 426 15271161 10.1111/j.1365-2648.2004.03123.x

[25] Rahtz E Bhui K Hutchison I Korszun A Are facial injuries really different? An observational cohort study comparing appearance concern and psychological distress in facial trauma and non-facial trauma patients J Plast Reconstr Aesthet Surg 2018 71 01 62 71 28935194 10.1016/j.bjps.2017.08.006

[26] Chang C-M Ko E C Kao C-C Chang P-Y Chen M YC Incidence and clinical significance of zygomaticomaxillary complex fracture involving the temporomandibular joint with emphasis on trismus Kaohsiung J Med Sci 2012 28 06 336 340 22632890 10.1016/j.kjms.2011.11.016 PMC11916773

[27] Yun P-Y Kim Y-K The role of facial trauma as a possible etiologic factor in temporomandibular joint disorder J Oral Maxillofac Surg 2005 63 11 1576 1583 16243173 10.1016/j.joms.2005.05.318

[28] Kommers S C van den Bergh B Boffano P Verweij K P Forouzanfar T Dysocclusion after maxillofacial trauma: a 42 year analysis J Craniomaxillofac Surg 2014 42 07 1083 1086 23849246 10.1016/j.jcms.2013.05.013

